# One-Step Preparation of Ion-Exchangeable Biochar for Enhanced Pb (II) Adsorption

**DOI:** 10.3390/molecules31091399

**Published:** 2026-04-23

**Authors:** Zhangshuai Ding, Hao Sun, Yujia Wu, Defa Hou, Xu Lin, Fulin Yang, Yunwu Zheng, Can Liu

**Affiliations:** National Joint Engineering Research Center for Highly-Efficient Utilization Technology of Forest Biomass Resources, Southwest Forestry University, Kunming 650224, China

**Keywords:** pyrolysis, agricultural and forestry waste, biochar, lead, adsorption

## Abstract

The safety of drinking water has a significant impact on human life and health, with the common presence of Pb (II) causing harm to human beings. The physical adsorption method is an effective means of removing Pb (II) from water. In this study, three types of biochar were produced through a one-step process using agricultural and forestry wastes (rape straw, bagasse, and walnut shell) as raw materials and KHCO_3_ as a co-carbonization agent. The resulting biochar exhibited remarkable adsorption capacities for Pb (II). The biochar prepared via a single carbonization process demonstrates excellent adsorption performance towards Pb (II). The adsorption capacity of bagasse-derived biochar reaches 76.94 mg/g, which is 4.5-fold higher than that of the control. For walnut shell-derived biochar, the adsorption value attains 124.90 mg/g, representing a 7.5-fold enhancement. Notably, rape straw-derived biochar demonstrates the maximum adsorption capacity, up to 265.69 mg/g. Mechanistic analysis reveals that the adsorption of rape straw biochar is dominated by ion exchange, while also being influenced by physical adsorption, coprecipitation, and electrostatic attraction. Intriguingly, in this study, the sole use of KHCO_3_ as a co-carbonization agent remarkably increases the specific surface area of the biochar and facilitates the formation of micropores. Without the need for pre-carbonization, this approach substantially boosts the Pb (II) adsorption capacity of the biochar. This one-step carbonization strategy exhibits distinct operational convenience and cost-effectiveness, providing promising materials for the low-cost removal of Pb (II) in natural water bodies and open environments, while also offering a viable technical route for the fabrication of high-performance biochar for heavy metal remediation.

## 1. Introduction

Water is the fundamental source of life, and pollution poses a significant threat to human health [1]. With the advancement of industry, the ecological environment has been compromised, leading to increasingly severe lead contamination [2]. Lead can infiltrate the human body through the food chain, causing substantial harm to the nervous system, cardiovascular system, and immune system, particularly impacting children [3]. Waterborne lead pollution commonly occurs in agricultural irrigation water, mining water sources, and factory discharge effluents [4,5]. According to the World Health Organization’s report, the Pb concentration in drinking water should not exceed 0.01 mg/L. Therefore, eliminating heavy metal Pb from water bodies holds great significance for enhancing human health and promoting children’s development [3].

There are many methods for removing lead ions in water, such as precipitation, electrolysis, ion exchange and adsorption. Physical adsorption method has the advantages of convenient operation, high efficiency, low energy consumption and no secondary pollution to water bodies, and is often used as a method to treat heavy metals in water bodies [6]. At present, there are many types of materials that can be used as adsorbents to remove heavy metals from water, including synthetic materials, clay minerals, natural zeolites, biochar, etc. Among these materials, biochar has attracted widespread attention due to its low cost, extensive availability, and feasibility of being prepared from agricultural and forestry wastes [7]. Biochar has become a popular adsorbent material due to its wide source of raw materials and renewable and abundant surface functional groups. However, the adsorption capacity of biochar is limited, and in order to improve the adsorption effect of biochar, biochar is usually modified. Wenchao Yang et al. used Enterobacter derivatives as raw materials to prepare biochar and conducted adsorption studies on Cu (II) and Pb (II) in seawater, and found that the prepared biochar had a high removal rate for both of them. Within the adsorption time of 60 min, the removal rates of Cu (II) and Pb (II) reached 91% and 54%, respectively [8]. Huayi Chen et al.’s chicken feather activated carbon prepared with KOH as activator adsorbed Cd (II) and Pb (II) in water, and the adsorption capacity reached 7.07 times and 26.52 times of the original carbon, respectively [9]. Through modification, the adsorption capacity of biochar has been greatly improved.

In recent years, the production of agriculture and forestry industry has increased sharply, and the waste generated has caused a burden to the environment. Scholars have begun to pay attention to the efficient utilization of agricultural and forestry waste [10,11,12]. The preparation of biochar from waste agricultural and forestry biomass is one of the effective ways of utilization. This treatment method not only increases the added value of agriculture and forestry industry, but also contributes to environmental protection [13]. Hirpo Hinsene et al. doped low-eutectic solvent and Fe_3_O_4_/ZnO nanoparticles with rice husks as raw materials to prepare biomass activated carbon, which was used to remove Cr (VI), Pb (II) and diclofenac sodium from water bodies, and the maximum adsorption capacities were 66.23, 384.62 and 24.33 mg/g, respectively [14]. Different pyrolysis methods, pyrolysis parameters and raw material selection are all important parameters affecting the physicochemical properties, structure and heavy metal ion removal capacity of biochar [15]. The biochar derived from a simple carbonization process exhibits relatively low adsorption capacity. For example, Ye et al. prepared biochar via pyrolysis carbonization of cow manure and cherry wood as raw materials at high temperatures of 400 °C, 500 °C, and 600 °C, respectively. The experimental results demonstrated that the maximum Pb (II) adsorption capacity of the biochar CMB6 was merely 40.8 mg/g [16]. Emenike et al. conducted co-carbonization of bean shells with LDPE (low-density polyethylene), and the resulting biochar showed maximum adsorption capacities of 36.63 mg/g for Cr (VI) and 15.22 mg/g for Pb (II), respectively [17]. Simić et al. modified corn cob biochar through a two-step conversion process. Firstly, they prepared corn cob biochar by hydrothermal carbonization, and then doped MgCl_2_ with the corn cob biochar and carried out pyrolysis. The maximum adsorption capacity of the synthesized hydrothermal pyrolysis carbon (HCC-Mg) for Pb (II) was 87.08 mg/g [18]. Similarly, Ercegović et al. also synthesized hydrothermal pyrolysis carbon biochar (C-HPCM) through a two-step conversion method involving hydrothermal carbonization followed by pyrolysis, achieving a maximum adsorption capacity of 155.6 mg/g for Pb (II) [19]. Using KOH as the activating agent, Zhu et al. synthesized activated carbon via hydrothermal carbonization using municipal sludge as the biomass feedstock, whereas Hu et al. prepared activated carbon through pyrolysis with coffee grounds as the biomass raw material. The adsorption capacities of the activated carbons thus obtained for Pb (II) were 48.6 mg/g and 428.6 mg/g, respectively, representing a nearly 100-fold difference in adsorption performance [20,21]. To summarize, modifying biochar can significantly enhance its adsorption performance for metal ions; however, the preparation process typically involves two or more steps. Such modification protocols impose substantial cost burdens on the remediation of metal ions in natural water bodies and open environments.

**Figure 1:**
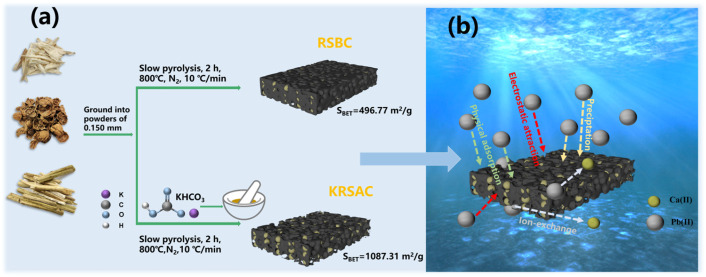
(**a**) Flowchart of the biochar preparation route; (**b**) mechanism of Pb (II) removal.

In this study, a highly simplified biochar preparation process is proposed, which enables the production of biochar with high Pb (II) adsorption performance via a single carbonization step (Figure 1). This process significantly enhances the specific surface area of the resulting biochar and substantially reduces the cost of biochar for Pb (II) adsorption, thereby exhibiting considerable potential for Pb (II) removal applications in natural water bodies and open environments. Thus, the low-cost removal of Pb (II) can be realized in natural water bodies and open environments, exhibiting significant potential for practical applications.

## 2. Results

### 2.1. Characteristics of Carbon Before and After Activation

#### 2.1.1. Pore Structure Analysis

**Figure 2:**
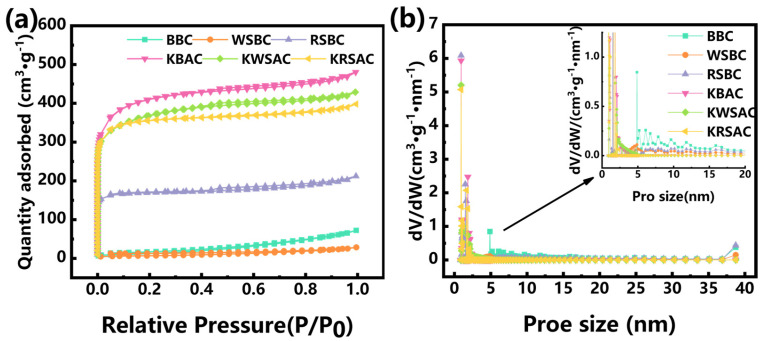
(**a**) N_2_ adsorption–desorption isotherm curves of biochar before and after activation; (**b**) pore size distribution of biochar before and after activation.

The adsorption isotherms of BBC (bagasse biochar), WSBC (walnut shell biochar), RSBC (rape straw biochar), KBAC (KHCO_3_-co-carbonized sugarcane bagasse biochar), KWSAC (KHCO_3_-co-carbonized walnut shell biochar), and KRSAC (KHCO_3_-co-carbonized rape straw biochar) conform to the Type I and Type II composite models classified by IUPAC [22]. They belong to the mixed type of microporous and mesoporous activated carbon, and have an H4 hysteresis loop. The pore structures of BBC, WSBC, and RSBC are predominantly mesoporous and macro-porous, with average pore sizes ranging from 2.50 nm to 8.01 nm. Upon co-carbonization with KHCO_3_, the pore sizes of the three resultant biochar (KBAC, KWSAC, and KRSAC) were significantly reduced to 2.2 nm (Figure 2b and Table 1). This distinctive micro-mesoporous architecture enhances the specific surface area of the biochar, with all three co-carbonized biochar exhibiting a specific surface area exceeding 1000 m^2^/g (Table 1). Biochar with high specific surface area often exhibits high adsorption capacity [23]. Analysis reveals that potassium vapor generated by the decomposition of KHCO_3_ at elevated temperatures contributes to the formation of a rich pore structure on the biochar surface. The unique structure of the co-carbonized biochar prepared in this study provides a favorable structural foundation for subsequent adsorption applications. Thus, a high specific surface area can be achieved via our single-step carbonization process.

#### 2.1.2. SEM Analysis of Apparent Morphology

**Figure 3:**
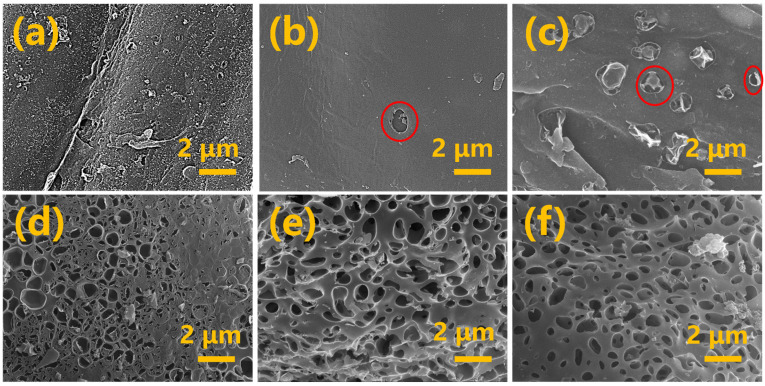
The SEM images of biochar before and after activation: (**a**) BBC; (**b**) WSBC; (**c**) RSBC; (**d**) KBAC; (**e**) KWSAC; (**f**) KRSAC.

To further investigate the effect of biochar’s microstructure on its adsorption efficiency, scanning electron microscopy (SEM) was employed in this study for observational analysis. The study found that the three types of raw biomass char (BBC, WSBC, and RSBC) exhibit relatively few surface pores, with only RSBC showing a small number of pores (Figure 3a–c), which is consistent with the biochar pore size analysis data (Table 1). The addition of co-carbonization agents can significantly increase the number of pores on the biochar surface. Compared with the other two types of biochar, the pore distribution on the surface of KRSAC is more abundant and uniform (Figure 3d–f). These pores increase the specific surface area of the biomass char and provide a large number of active sites for Pb (II) adsorption [24].

#### 2.1.3. XRD, FTIR and Raman Analysis of Biochar

**Figure 4:**
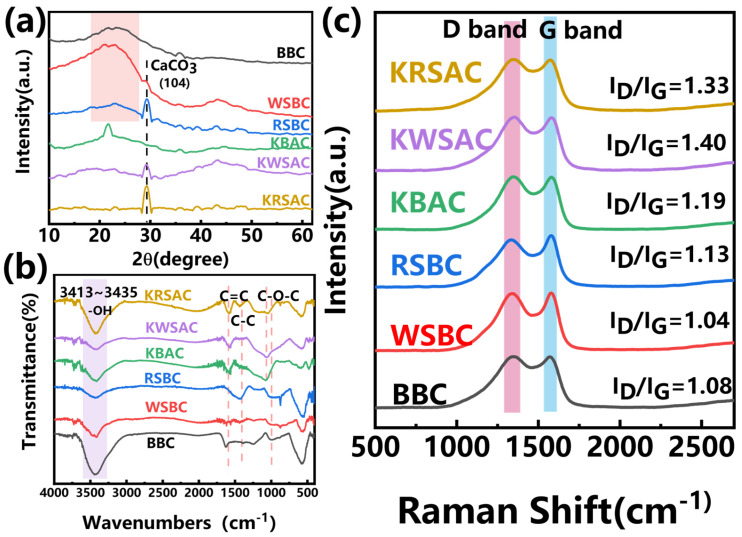
(**a**) XRD pattern of carbon before and after activation; (**b**) FT-IR spectra of carbon before and after activation; (**c**) Raman spectra of carbon before and after activation.

The XRD spectrum (Figure 4a) shows that there are differences among the three types of biochar. Both WSBC and RSBC exhibit CaCO_3_ (PDF#47-1743) characteristic diffraction peaks at 2θ = 29° [25]; the peak of RSBC is stronger than WSBC. There was no obvious crystallization peak observed in BBC, and a disordered carbon diffraction peak appeared at 20–30° [26]. Biochar forms a rich pore structure on the surface due to the effect of potassium vapor decomposed by KHCO_3_ at high temperatures; at the same time, it also exposed more CaCO_3_ (Figure 4a). The CaCO_3_ crystallization peaks of KWSAC and KRSAC are significantly enhanced, while KBAC shows a relatively strong SiO_2_ crystallization peak at 2θ = 22°, indicating that KHCO_3_ activation increased the crystallinity of biochar, because the reaction process was intense, exposing the mineral elements within the biomass and forming crystals (Figure 4a).

The FT-IR spectrum (Figure 4b) shows that the surface functional groups of biochar before and after activation are similar, with oxygen-containing and aromatic functional groups dominating [27]. The peak at 3200–3500 cm^−1^ is related to O-H stretching vibration, while the peak at 1020–1070 cm^−1^ is related to C-O stretching vibration [28]. The peaks at 1580 cm^−1^ and 1440 cm^−1^ are caused by the C=C stretching vibration and C-C skeleton vibration in the aromatic structure [29]. The addition of co-carbonization agents does not induce modification of the surface functional groups of biochar.

To further characterize the defect degree of biochar, Raman spectroscopy measurements were performed on the samples. The addition of co-carbonization agents resulted in an increase in the ID/IG ratio, indicating that the graphitized structure of biochar was disrupted and the number of defects was enhanced (Figure 4c). The increased proportion of defect states and the exposure of more adsorption sites facilitate the removal of lead ions.

### 2.2. Analysis of Pb (II) Adsorption Behavior

**Figure 5:**
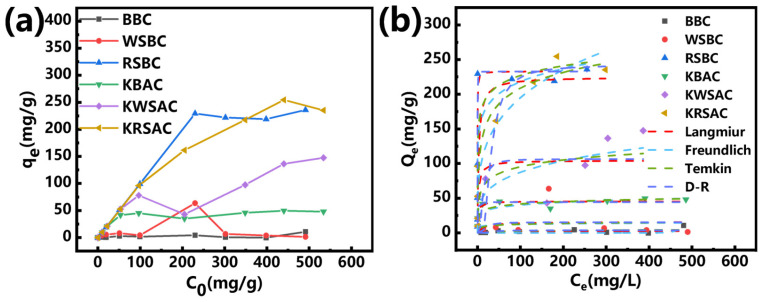
(**a**) The effect of initial concentration on the adsorption capacity of biochar; (**b**) fitting curve of adsorption isotherm model.

This study prepared three types of biochar through a very simple co-carbonization experiment to adsorb Pb (II) in water. The adsorption performance of KHCO_3_-co-carbonized biochar toward aqueous Pb (II) exceeds that of pristine biomass biochar (Figure 5a). Rape straw-derived biochar exhibits superior Pb (II) adsorption performance compared to sugarcane bagasse-derived and walnut shell-derived biochar. It has been confirmed that the co-pyrolysis strategy involving the addition of KHCO_3_ as a co-carbonization agent can effectively enhance the heavy metal Pb (II) removal capacity of biomass biochar [30].

From the perspective of the correlation of the fitting models (Table 2), the fitting results of the BBC and WSBC models are not ideal, which is related to their small adsorption capacity and poor adsorption effect (Figure 5a). The fitting results of the four models (RSBC, KBAC, and KRSAC) all have a relatively high correlation. Meanwhile, the experimental results show that the actual maximum adsorption capacities of the three biochars (RSBC, KBAC, and KRSAC) are 236.028 mg/g, 49.62 mg/g, and 254.56 mg/g, respectively, which are close to the fitting results of the Langmuir model and the Dubinin–Radushkevich model. This indicates that the adsorption process of the three biochar for Pb (II) is rather complex and influenced by multiple factors. The Freundlich model of KWSAC has a higher fitting correlation, suggesting that its adsorption process is more in line with multilayer adsorption [31]. The adsorption behavior of lead ions on biochar (KRSAC) involves a complex adsorption mechanism.

**Figure 6:**
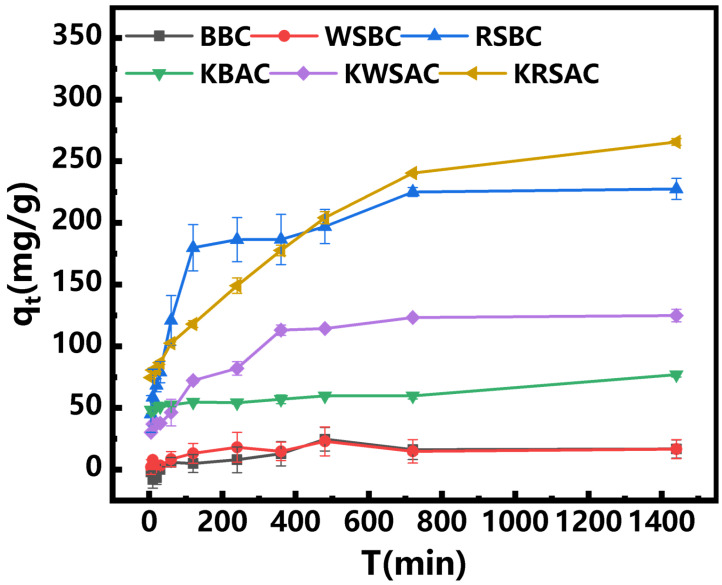
The effect of adsorption time on the adsorption capacity of biochar.

In order to study the effect of adsorption time on the adsorption capacity of biochar, a time gradient experiment was conducted, and the experimental results are shown in Figure 6. As adsorption time increased, the adsorption capacity of biochar increased accordingly. At 360 min, the adsorption capacities of the three pristine biochar (BBC, WSBC, and RSBC) reached a plateau, whereas KBAC, KWSAC, and KRSAC did not stabilize until 720 min. This phenomenon indicates that the adsorption sites on the surfaces of BBC, WSBC, and RSBC are limited, failing to continuously provide effective binding sites for Pb (II). When the initial concentration of the solution was 400 mg/L, the maximum adsorption capacity of RSBC for Pb (II) was 227.54 mg/g, and under the same conditions, the adsorption capacity of KRSAC for Pb (II) reached 265.69 mg/g. The Pb (II) removal efficiency of the biochar followed the order KRSAC > RSBC > KWSAC > WSBC > KBAC > BBC.

**Figure 7:**
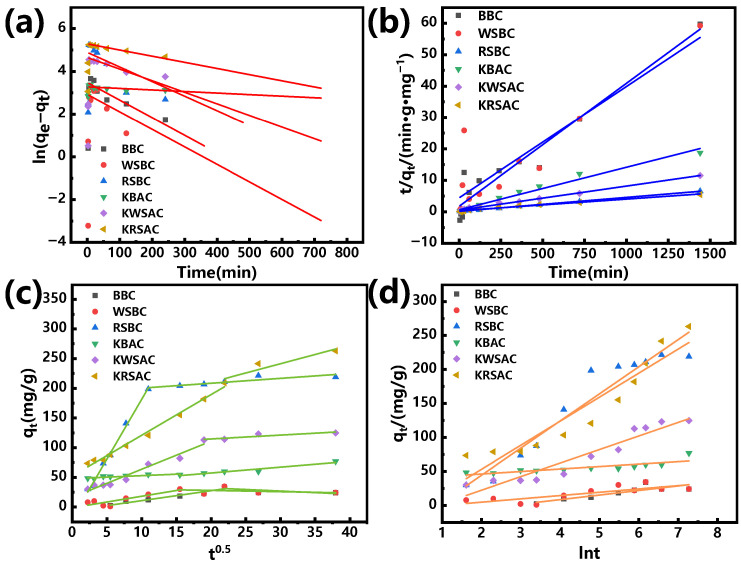
Dynamic model fitting curve: (**a**) pseudo-first-order dynamic model; (**b**) pseudo-second-order dynamic model; (**c**) intraparticle diffusion model; (**d**) Elovich model.

As shown in Figure 7 and Table 3, the researchers respectively applied the pseudo-first-order kinetic model equation, the pseudo-second-order kinetic model equation, the intraparticle diffusion model equation and the Elovich model equation to fit the experimental data, aiming to explore the adsorption rate, kinetic characteristics and diffusion mechanism of biochar.

According to the fitting results in Table 3, the pseudo-first-order kinetic model of BBC and WSBC has a higher correlation, indicating that their adsorption behavior is more inclined towards physical adsorption, mainly controlled by diffusion, and the adsorption capacity is affected by the adsorption sites on the surface of the biochar. Combined with the analysis of the XRD pattern (Figure 4a), Ca (II) exists on the surface of RSBC and it has a larger specific surface area (Table 1), which is conducive to the ion exchange of Pb (II). Therefore, the pseudo-second-order kinetic model of RSBC has a higher fitting correlation, indicating that its adsorption behavior is mainly chemical adsorption. As shown in Figure 4a,c, after co-carbonization with KHCO_3_, the graphitization degree of KBAC and KWSAC decreased, and regular crystalline peaks (SiO_2_ and CaCO_3_) were formed on the surface, providing conditions for the ion exchange of Pb (II). Therefore, the fitting degree of the pseudo-second-order kinetic model of the two is higher. The difference in the fitting degree of the pseudo-first-order and pseudo-second-order kinetic models of KRSAC is small, at 0.005, indicating that the adsorption behavior of KRSAC involves both physical and chemical reactions. The intraparticle diffusion model is mainly used to describe the complex multi-step process in adsorption. The fitting piecewise linearity of the six biochar internal diffusion models did not pass through the origin, indicating that particle internal diffusion is not the only way to control adsorption behavior, and there are other influencing factors (Figure 7c). The Elovich model is used to describe a heterogeneous diffusion mode of Pb on the surface of biochar. At an initial concentration of 400 mg/L, there was a significant difference in the initial adsorption rate of biochar. The KWSAC and KRSAC showed a significant increase in the adsorption rate of Pb (II). This may be related to ion exchange and multilayer adsorption behavior [32].

### 2.3. Characteristics of Activated Carbon Before and After Adsorption

#### 2.3.1. XRD and FTIR Analysis of Activated Carbon

**Figure 8:**
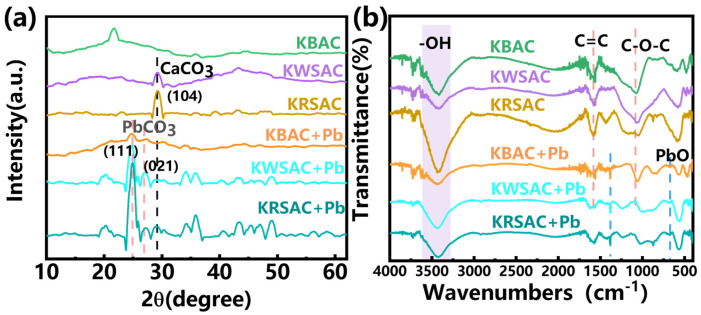
(**a**) XRD pattern of carbon before and after adsorption; (**b**) FT-IR spectra of carbon before and after adsorption.

Analysis of the post-adsorption spectra (Figure 8a) shows that characteristic diffraction peaks corresponding to PbCO_3_ (PDF#70-2052) emerge at 2θ = 24.5°, 25.6°, and 36.1° on the surface of all three biomass activated carbons following adsorption [33]. The intensity of the PbCO_3_ characteristic peak for KWSAC and KRSAC biochar was significantly higher than that of KBAC (Figure 8a). This observation indicates that KWSAC and KRSAC exhibit a greater capacity for Pb (II) adsorption, which in turn results in a higher intensity of the PbCO_3_ characteristic peak in the biochar. The superior Pb (II) adsorption performance of the two biochar may be attributed to the higher CaCO_3_ content in their respective precursor biochar (Figure 4a). This suggests that during the adsorption process, Ca (II) undergoes ion exchange with Pb (II), leading to the formation of PbCO_3_ precipitates—owing to the fact that the solubility product constant (Ksp) of PbCO_3_ is substantially lower than that of CaCO_3_. The high Ca (II) content enables ion exchange with Pb (II), thereby conferring KRSAC with a higher PbCO_3_ adsorption capacity [34]. Notably, the intensity of the PbCO_3_ diffraction peak in the post-adsorption KRSAC spectrum is the highest (Figure 8a).

The FTIR spectra show that the peak of the C=C bond at 1580 cm^−1^ of KRSAC decreased, and the C-O-C bond at 1070 cm^−1^ shifted (Figure 8b) after adsorption, indicating that a complexation reaction occurred during the adsorption process [35]. Moreover, characteristic peaks of PbO appeared near 675 cm^−1^ for all three types of activated carbon after adsorbing Pb (II).

#### 2.3.2. SEM-EDS Analysis of Apparent Morphology

**Figure 9:**
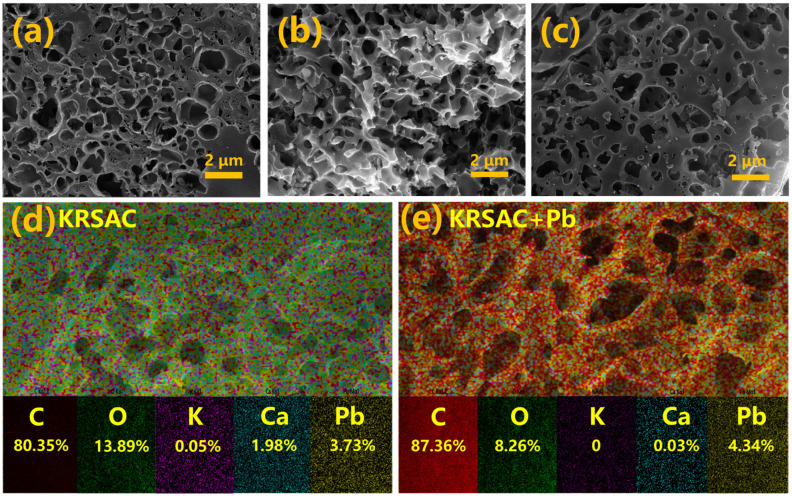
(**a**) The SEM images of KBAC after adsorption; (**b**) the SEM images of KWSAC after adsorption; (**c**) the SEM images of KRSAC after adsorption; (**d**) the EDS of KRSAC; (**e**) the EDS of KRSAC after adsorption.

Electron microscopy observations and energy dispersive X-ray spectroscopy (EDS) analyses of the three biochar samples before and after adsorption indicated that the pore structure on the surface of the biochar remained largely intact after adsorbing Pb (II), but the roughness of the pore edges increased (Figure 9a–c). According to the EDS data analysis of the biochar (Figure S4), the contents of oxygen (O) and calcium (Ca) in KBAC, KWSAC, and KRSAC followed the order of KRSAC > KWSAC > KBAC. After adsorption, the contents of O and Ca in the biochar decreased, while the content of Pb increased, indicating that Pb was effectively adsorbed and ion exchange occurred during the adsorption process [17]. The EDS data analysis further confirmed the higher initial content of calcium carbonate (CaCO_3_), which provided a basis for the adsorption of Pb (II)—consistent with the trend observed in Figure 8a. As shown in Figure 9a–c, the surface of the activated carbon after adsorption was significantly rougher than before adsorption, with small crystalline particles formed. The adsorption of Pb on the surface of the biochar was relatively uniform (Figure 9e). Additionally, KRSAC contained higher amounts of Ca and O, theoretically enabling it to adsorb more Pb (II) (Figure 9d, e). Before adsorption, the contents of O and Ca in KRSAC were 13.89% and 1.98%, respectively, and after adsorption, they decreased to 8.26% and 0.03%, respectively, while the content of Pb increased to 4.34%. This is consistent with the final adsorption performance results and once again confirms that the adsorption behavior of KRSAC is mainly based on ion exchange reactions [36].

#### 2.3.3. XPS Analysis of Activated Carbon Before and After Adsorption

**Figure 10:**
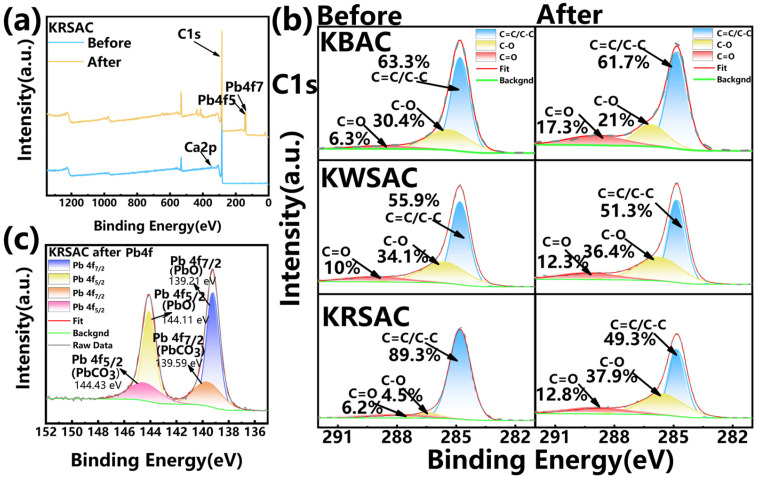
(**a**) Total XPS spectrum before and after KRSAC adsorption; (**b**) peak profiles of C1s before and after KBAC, KWSAC and KRSAC adsorption; (**c**) Pb peak spectra after KRSAC adsorption.

The XPS spectrum (Figure 10) is used to further analyze the surface element composition and chemical valence state of activated carbon before and after adsorption. From the total spectrum, it can be seen that in addition to the C1s peak, the KRSAC before adsorption also exhibits a Ca2p characteristic peak at around 351 eV (Figure 10a). After adsorption, there are obvious characteristic peaks of PbCO_3_ and PbO at 144 eV (Figure 10c) [37], further indicating the presence of ion exchange during the adsorption process. Comparing the C1s peaks before and after adsorption, it can be seen that the C=C/C-C bond content of the three activated carbons decreased after adsorption. KBAC decreased from the original 63.3% to 61.7%, KWSAC decreased from 55.9% to 51.3%, while KRSAC had the most significant change, from 89.3% before adsorption to 49.3%, indicating that π-π bonds in the aromatic ring are involved in the adsorption process (Figure 10b). The C=O bond content of the three activated carbons increased after adsorption, while the C-O bond content showed a decrease in KBAC and an increase in KWSAC and KRSAC, indicating that the adsorption mechanism of KRSAC is different from that of KBAC and KWSAC. During the adsorption process of KRSAC, not only ion exchange occurs, but also other reactions occur [25,38,39].

## 3. Conclusions

In summary, this study synthesized high-specific-surface-area biochar via a one-step method using sugarcane bagasse, walnut shells, and rapeseed straw as feedstocks and potassium bicarbonate (KHCO_3_) as a co-carbonization agent, which was employed for the adsorption of Pb (II) in aqueous solutions. Based on adsorption capacities, the order observed was KRSAC > RSBC > KWSAC > KBAC. The co-carbonization agent KHCO_3_ effectively increased the specific surface area of the biochar, and the pore structure of the co-carbonized biochar was predominantly microporous. While KHCO_3_ enhanced the adsorption capacity of biochar, its improvement effect was limited for rapeseed straw-derived biochar, whereas the enhancement for walnut shell-derived biochar was the most significant. Among the three biomass feedstocks, the KRSAC biochar prepared from rapeseed straw exhibited the highest adsorption capacity, with a maximum Pb (II) adsorption capacity of 265.69 mg/g in aqueous solutions. Analysis revealed that the adsorption mechanism of rape straw biochar was dominated by ion exchange reactions in chemical adsorption, with additional contributions from electrostatic adsorption, physical adsorption, and coprecipitation [7]. This technology features low energy consumption and high efficiency, offering a novel technical route for the fabrication of high-performance biochar.

## 4. Materials and Methods

### 4.1. Experimental Materials

Bagasse was taken from a fruit store in Binchuan, Dali, Yunnan, China; walnut shell was provided by a processing plant Yangbi, Dali, Yunnan, China. Rape straw was purchased from Luoping farmers’ market. The three raw materials, sugarcane bagasse, walnut shell, and rapeseed straw, were washed with water and dried at 100 °C for 12 h with a moisture content of less than 7%. The biomass was then crushed, passed through a 100-mesh stainless steel mesh sieve, and sealed for storage for later use.

The reagents and drugs used in the experimentation were all analytical pure grade, such as KHCO_3_, Pb (NO_3_)_2_, HNO_3_, NaOH and oleic acid. In addition, the water used during the experiment was all ultrapure water.

### 4.2. Preparation of Biochar

Preparation of Biochar: 5 g of biomass was accurately weighed and subjected to pyrolysis in a tube furnace (OTF-1200X, Kejing Instruments, Hefei, China). The furnace was heated at a rate of 10 °C min^−1^ to 800 °C and held at this temperature for 2 h, followed by natural cooling to room temperature. The resulting biochar was then rinsed with ultrapure water until the effluent reached neutral pH, dried at 80 °C for 12 h, and labeled separately as BBC (bagasse-derived biochar), WSBC (walnut shell-derived biochar), and RSBC (rapeseed straw-derived biochar). The samples were hermetically sealed and stored for subsequent use.

Preparation of Co-carbonized Biochar: The preparation procedure for co-carbonized biochar is analogous to that of biochar. Specifically, 5 g of biomass and 5 g of potassium bicarbonate (KHCO_3_) were accurately weighed and thoroughly ground and mixed in a ceramic mortar. The mixture was then transferred into a tube furnace. The pyrolysis conditions and subsequent treatment steps were identical to those detailed in the preceding biochar preparation protocol, with the critical caveat that the nitrogen (N_2_) flow must remain continuous and uninterrupted throughout the entire process. The resulting co-carbonized biochar samples were respectively designated as KBAC (KHCO_3_-co-carbonized sugarcane bagasse biochar), KWSAC (KHCO_3_-co-carbonized walnut shell biochar), and KRSAC (KHCO_3_-co-carbonized rapeseed straw biochar), and were hermetically sealed for subsequent experimental use.

### 4.3. Methods

In order to study the influence of the physicochemical properties of biochar on its adsorption capacity, the N_2_ adsorption and desorption isotherms of biochar were measured using a physical adsorption instrument (ASAP 2020, Thermo Fisher, Waltham, MA, USA), and the specific surface area of biochar was calculated using the BET method. Scanning electron microscopy (Mira 4, Tescan, Brno, Czech Republic) was used to analyze the surface morphology and microstructure of biochar. The phase structure of the biochar was analyzed using an X-ray diffractometer (Ultima, Rigaku, Tokyo, Japan). The degree of graphitization of biochar before and after activation was characterized by Raman spectroscopy (HORIBA Scientific LabRAM HR Evolution, HORIBA Scientific, Villeneuve d’Ascq, France). FT-IR (IS50, Thermo Fisher, Waltham, MA, USA) was used to analyze the functional group composition of biochar before and after activation and adsorption, while XPS (Scientific K-Alpha, Thermo Fisher, Waltham, MA, USA) was used to determine the chemical state changes of surface elements of biochar before and after adsorption. All adsorbed samples were measured using ICP-OES (VISTA-MPX, Varian, Palo Alto, CA, USA).

### 4.4. Study on the Adsorption Performance of Activated Carbon

#### 4.4.1. Adsorption Experiments

1. The influence of initial concentration and adsorption time.

Firstly, a 1000 mg/L Pb (II) standard stock solution was prepared. Subsequently, it was serially diluted to seven sets of standard working solutions with concentration gradients ranging from 10 to 500 mg/L in accordance with experimental requirements. In the adsorption experiment, 0.03 g of biochar was added to 30 mL of Pb (II) solution. The adsorption experiment was conducted at 25 °C and a constant shaking table speed of 300 rpm. The adsorption time effect experiment was conducted at a total of 11 time points between 5 and 1440 min. The initial concentration of Pb (II) solution was 400 mg/L, and the temperature was 25 °C. The adsorption capacity of Pb (II) is calculated according to the following formula:

(1) $$q_{e}=\frac{C_{0}{-C}_{e}}{m}\times V$$

(2) $$q_{t}=\frac{C_{0}{-C}_{t}}{m}\times V$$

where C_0_, Ce, and Ct are the initial concentration of the solution, the concentration at adsorption equilibrium, and the concentration of the solution at a certain moment re, mg/L, respectively; V is the volume of the adsorption solution, L; q_e_ and q_t_ are the adsorption capacity of the adsorbent at equilibrium and the adsorption capacity of the adsorbent at a certain moment, mg/g, respectively. And m is the amount of biochar, g.

2. Other interfering factors (pH, the amount of biochar, temperature).

In addition to experiments on the effects of initial concentration and adsorption time, other related factors such as solution pH, amount of biochar added, and temperature were also studied. Using a 0.1 mol/L HNO_3_ solution and NaOH solution to adjust the pH value of a Pb (II) solution with an initial concentration of 400 mg/L, the pH values of the Pb (II) solution are 1.24, 2.39, 3.15, 3.64 and 4.94 respectively. An adsorption experiment was conducted. The adsorption time was 24 h and the temperature was 25 °C. The initial concentration of Pb (II) solution was 400 mg/L, and adsorption experiments were conducted at 25 °C, 35 °C, and 45 °C water bath conditions for 6 h. The experiment on the dosage of biochar was conducted at an initial concentration of 400 mg/L. The biochar was added to 30 mL of Pb (II) solution at solid–liquid ratios of 0.5 g/L, 1 g/L, 1.5 g/L, 2 g/L, and 2.5 g/L. The experimental results and data can be found in the Supplementary Materials.

#### 4.4.2. Adsorption Isotherm Model

Adsorption isotherms are used to assess the suitability of the adsorption process and to evaluate the maximum adsorption capacity of the adsorbent material. In addition, the fit of the isotherm model to the experimental data provides information about the adsorption properties. This article selected four models to fit the adsorption data, the Langmuir model, Freundlich model, Temkin model and Dubinin–Radushkevich model.

(1) Langmuir isotherm model.

The expression is as follows:

(3) $$Q_{e}=\frac{Q_{max}K_{L}C_{e}}{1\;+\;K_{L}C_{e}}$$

where Q_e_—equilibrium adsorption capacity (mg/g); K_L_—the Langmuir constant related to the heat of adsorption (L/mg); C_e_—equilibrium concentration (mg/L); and Q_m_—maximum adsorption capacity of the adsorbent (mg/g).

(2) Freundlich isotherm model.

The expression is as follows:

(4) $$Q_{e}=K_{F}{C_{e}}^{\frac{1}{n}}$$

where Q_e_—equilibrium adsorption capacity (mg/g); K_F_—Freundlich adsorption isotherm constant; n—adsorption strength; and C_e_—equilibrium concentration (mg/L).

(3) Temkin isotherm model.

The expression is as follows:

(5) $$Q_{e}=\frac{RT}{b_{T}}\ln K_{T}\times C_{e}$$

where Q_e_—equilibrium adsorption capacity (mg/g); C_e_—equilibrium concentration (mg/L); R—gas constant (8.314 (J/mol·K); T—temperature (K); b_T_—Temkin constant (J·mol^−1^); and K_T_—Temkin isotherm constant (L/g).

(4) Dubinin–Radushkevich isotherm model.

The expression is as follows:

(6) $$Q_{e}=Q_{m}\;\times \;\exp(-K_{D}\times \varepsilon^{2})$$

where Q_e_—equilibrium adsorption capacity (mg/g); Q_m_—maximum adsorption capacity of the adsorbent (mg/g); K_D_—Dubinin–Radushkevich adsorption isotherm constant; and ε—Polanyi potential energy (J/mol).

#### 4.4.3. Adsorption Kinetics

Adsorption kinetics expresses the nature (physical or chemical) of the interaction dependence between adsorbate and adsorbent and is important for evaluating the mechanism and efficiency of the adsorption process.

(1) Pseudo-first-order kinetic equation.

The equation is expressed as follows:

(7) $$\ln(q_{e}-\;q_{t})=\ln q_{e}-\;k_{1}t\;$$

where q_e_ is the adsorption amount at equilibrium (mg/g); q_t_ is the adsorption amount at t (mg/g); k_1_ is the secondary rate constant (g/(mg·min^−1^); and t is the adsorption time (min).

(2) Pseudo-second-order kinetic equation.

The equation is expressed as follows:

(8) $$\frac{t}{q_{t}}=\frac{1}{k_{2}{q_{e}}^{2}}\;+\;\frac{t}{q_{e}}$$

where q_e_ is the adsorption amount at equilibrium (mg/g); q_t_ is the adsorption amount at t (mg/g); k_2_ is the secondary rate constant (g/(mg·min^−1^); and t is the adsorption time (min).

(3) Intraparticle diffusion equation.

The equation is expressed as follows:

(9) $$q_{t}=K_{\mathrm{id}}\sqrt{t}+C$$

where q_t_ is the adsorption amount at t (mg/g); k_id_ is the intraparticle diffusion rate constant (g/(mg·min^−1^); t is the adsorption time (min); and C is the intercept.

(4) Elovich equation.

The equation is expressed as follows:

(10) $$q_{t}=\frac{1}{\beta }\ln\left(\alpha \beta \right)\;+\;\frac{1}{\beta }\ln\left(t\right)$$

where q_t_ is the adsorption amount at t (mg/g); t is the adsorption time (min); α is the initial adsorption rate; and β is the desorption constant.

## Figures and Tables

**Table 1:** The parameters of N_2_ adsorption–desorption isotherm.

Sample	S_BET_ (m^2^/g)	S_micro_ (m^2^/g)	S_ext_ (m^2^/g)	V_total_ (cm^3^/g)	D_average_ (nm)
BBC	49.44	-	63.01	0.10	8.01
WSBC	24.49	4.57	19.92	0.04	6.14
RSBC	496.77	446.60	50.17	0.31	2.50
KBAC	1247.43	836.92	410.51	0.72	2.31
KWSAC	1157.56	743.71	413.85	0.65	2.24
KRSAC	1087.31	907.60	179.70	0.60	2.21

**Table 2:** Adsorption isotherm model parameters.

Isotherm Model		BBC	WSBC	RSBC	KBAC	KWSAC	KRSAC
Langmuir	Q_max_ (mg·g^−1^)	2.672	15.865	233.050	45.400	104.485	224.939
	K_L_ (L·g^−1^)	0.059	0.071	5.497	0.649	0.432	0.332
	R^2^	0.079	0.046	0.924	0.871	0.576	0.877
Frendlich	K_F_ (L·g^−1^)	4.357^−89^	7.748	125.906	21.772	27.558	55.666
	n	0.03	9.977	8.383	7.504	3.991	3.675
	R^2^	0.653	0.013	0.729	0.726	0.689	0.912
Temkin	b_T_ (J·mol^−1^)	2312.871	1429.678	122.774	517.931	175.817	75.606
	K_T_ (L·g^−1^)	0.122	13.144	764.094	60.533	8.692	5.826
	R^2^	0.199	0.019	0.780	0.782	0.654	0.915
Dubinin–Radushkevich	Q_max_ (mg·g^−1^)	3.678	15.268	232.734	44.311	106.559	242.240
	K_D_ (L·g^−1^)	920.980	271.080	0.204	1.643	119.402	789.674
	R^2^	0.149	0.049	0.946	0.872	0.453	0.810

**Table 3:** Dynamic model fitting parameters.

Adsorption Kinetics Model		BBC	WSBC	RSBC	KBAC	KWSAC	KRSAC
	q_m_(mg·g^−1^)	32.460	18.634	132.821	26.549	104.690	197.947
Pseudo-first-order	K_1_(min^−1^)	0.008	0.008	0.007	0.133	0.005	0.003
	R^2^	0.948	0.937	0.824	0.843	0.972	0.984
	q_m_(mg·g^−1^)	25.641	28.571	250.00	76.923	125.000	250.000
Pseudo-second-order	K_2_(g·mg^−1^·min^−1^)	0.001	0.000	0.000	0.000	0.000	0.000
	R^2^	0.929	0.788	0.999	0.976	0.992	0.979
	Kid_1_(mg/g·min^0.5^)	1.676	1.971	20.108	0.502	4.732	6.855
	C_1_	-	-	-	47.904	16.381	52.369
Intraparticle diffusion	R^2^	0.934	0.727	0.992	0.696	0.955	0.990
	Kid_2_(mg/g·min^0.5^)	-	-	0.822	0.981	0.648	3.120
	C_2_	42.778	32.331	192.083	37.765	101.729	147.746
	R^2^	0.128	-	0.744	0.898	0.693	0.783
	α	0.450	1.833	16.511	-	8.373	21.607
Elovich	β	0.149	0.206	0.025	0.274	0.050	0.028
	R^2^	0.711	0.620	0.915	0.665	0.904	0.883

## Data Availability

The original contributions presented in this study are included in the article/Supplementary Material. Further inquiries can be directed to the corresponding author.

## Supplementary Materials

The following supporting information can be downloaded at https://www.mdpi.com/article/10.3390/molecules31091399/s1. Figure S1: The influence of the amount of KRSAC; Figure S2: The influence of pH of KRSAC; Figure S3: The influence of temperature of KRSAC. Figure S4. The Graph of Element Content Variations in Biochar (KBAC, KWSAC and KRSAC) Before and After Adsorption.

## Abbreviations

The following abbreviations are used in this manuscript:

BBC	Bagasse biochar
WSBC	Walnut shell biochar
RSBC	Rape straw biochar
KBAC	KHCO_3_-co-carbonized sugarcane bagasse biochar
KWSAC	KHCO_3_-co-carbonized walnut shell biochar
KRSAC	KHCO_3_-co-carbonized rapeseed straw biochar
S_BET_	Specific surface area calculated by the BET method
S_micro_	Specific surface area of micropores
S_ext_	Specific surface area of extra pore categories
V_total_	Total pore volume
D_average_	Average pore diameter
