# Supplementary material for: One-Step Preparation of Ion-Exchangeable Biochar for Enhanced Pb (II) Adsorption
Source: Molecules. 2026 Apr 23;31(9):1399. doi: 10.3390/molecules31091399 (PMC13164738; doi:10.3390/molecules31091399)
Supplement: Supplementary file 1 [file molecules-31-01399-s001.zip › molecules-4230950-supplementary.pdf]

Supporting information

One-Step Preparation of Ion-Exchangeable Biochar for Enhanced Pb (II) Adsorption

Zhangshuai Ding , Hao Sun, Yujia Wu, Defa Hou, Xu Lin, Fulin Yang, Yunwu Zheng * and Can Liu*

National Joint Engineering Research Center for Highly-Efficient Utilization Technology of Forest Biomass Resources, Southwest Forestry University, Kunming 650224, China; dingzhangshuai99@163.com (Z.D.); wyj8099@163.com (Y.W.); houdefa001@163.com (D.H.); zyw85114@163.com (Y Z); linxunefu@126.com (X.L.); liucan@swfu.edu.cn (C.L.); yangfulin0309@163.com (F.Y.); sunhao@swfu.edu.cn (H.S.);

*Correspondence: zyw85114@163.com (Y Z); liucan@swfu.edu.cn (C.L.).

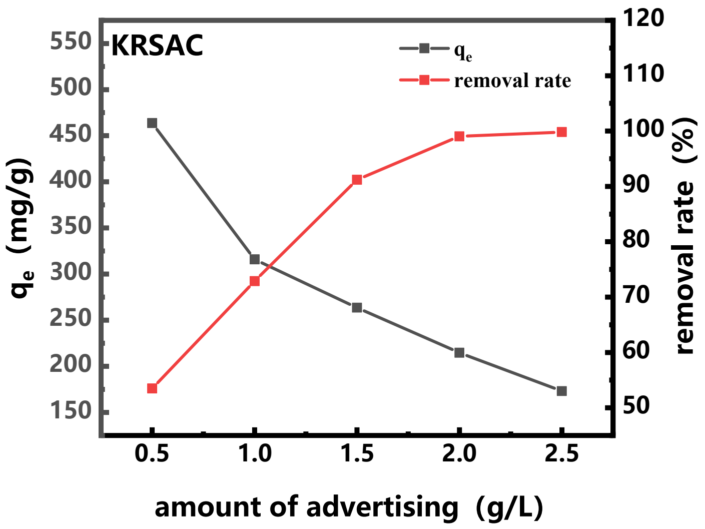

Figure S1. The influence of the amount of KRSAC

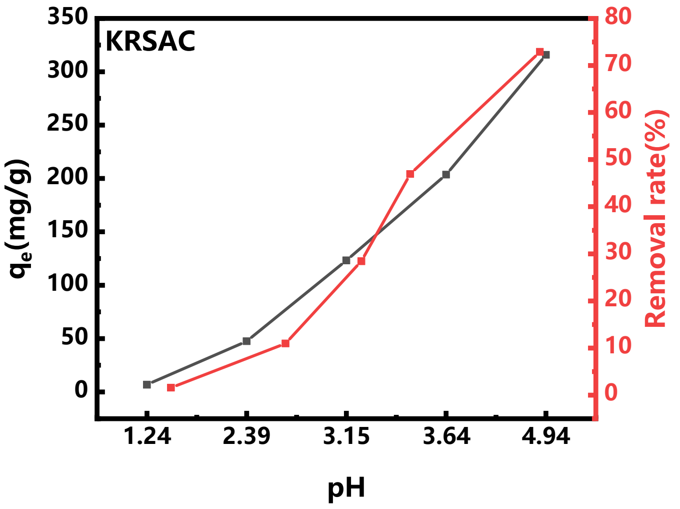

Figure S2. The influence of pH of KRSAC

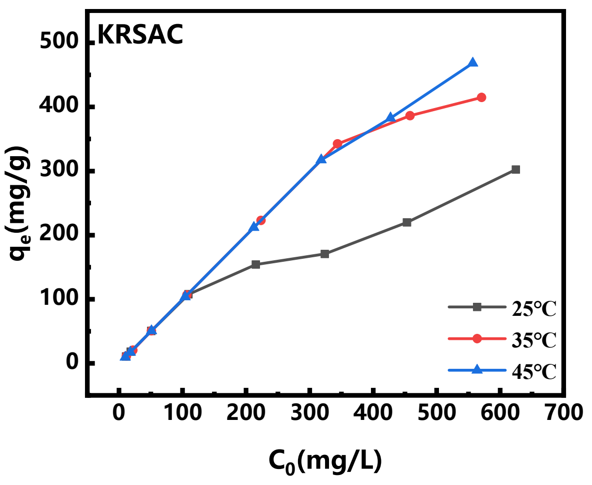

Figure S3. The influence of temperature of KRSAC

Figures S1, S2, and S3 all depict tests investigating the variation of KRSAC's adsorption capacity at an initial solution concentration of 400 mg/L. As shown in Figure S1, increasing the adsorbent dosage leads to a decrease in KRSAC's adsorption capacity alongside an increase in Pb (II) removal efficiency. However, these two parameters reach a relative equilibrium at an adsorbent dosage of 1 g/L, thus 1 g/L was adopted as the fixed dosage in all subsequent experiments.

Figure S2 reveals that both the adsorption capacity of KRSAC and the removal efficiency of Pb (II) increase with rising solution pH. Notably, during the experiment, white precipitates were observed when the solution pH exceeded 5, which was attributed to the influence of OH^- ions in the aqueous system. To avoid interference with the accurate determination of the activated carbon's adsorption capacity, all subsequent experiments were performed at a solution pH of 5.

Figure S3 presents the results of KRSAC's adsorption capacity experiments conducted at solution temperatures of 25°C, 35°C, and 45°C. It can be observed that higher solution temperatures facilitate the removal of Pb (II) by KRSAC. Considering that the temperature of natural water bodies is predominantly at room temperature, all subsequent experiments were carried out at 25°C.

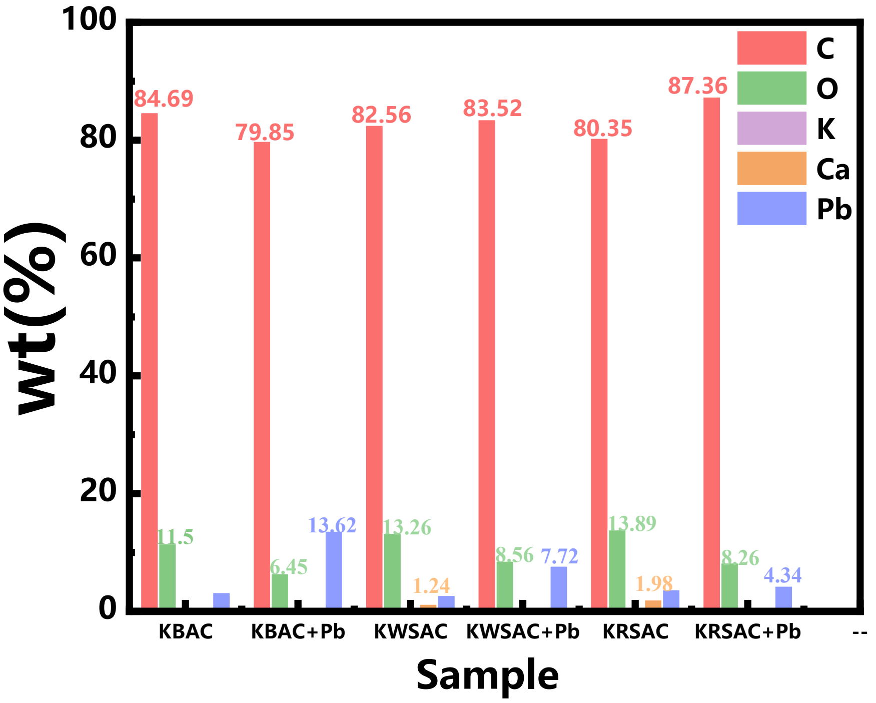

Figure S4. The Graph of Element Content Variations in Biochar (KBAC, KWSAC and KRSAC) Before and After Adsorption

Figure S4 is a summary graph based on EDS test data, recording the changes in elemental content (C, O, K, Ca, and Pb) of biochar (KBAC, KWSAC, and KRSAC) before and after adsorption. Generally speaking, the content of O and Ca decreased after adsorption, while the content of Pb increased, indicating that O and Ca participated in the reaction during the adsorption process.
